# Diagnosis and Management of Dysgerminomas with a Brief Summary of Primitive Germ Cell Tumors

**DOI:** 10.3390/diagnostics12123105

**Published:** 2022-12-09

**Authors:** Melinda-Ildiko Mitranovici, Diana Maria Chiorean, Maria Cezara Mureșan, Corneliu-Florin Buicu, Raluca Moraru, Liviu Moraru, Titiana Cornelia Cotoi, Ovidiu Simion Cotoi, Havva Serap Toru, Adrian Apostol, Sabin Gligore Turdean, Claudiu Mărginean, Ion Petre, Ioan Emilian Oală, Zsuzsanna Simon-Szabo, Viviana Ivan, Lucian Pușcașiu

**Affiliations:** 1 Department of Obstetrics and Gynecology, Emergency County Hospital Hunedoara, 14 Victoriei Street, 331057 Hunedoara, Romania; 2 Department of Pathology, County Clinical Hospital of Targu Mures, 540072 Targu Mures, Romania; 3 Department of Obstetrics and Gynecology, “Victor Babes” University of Medicine and Pharmacy, 2 Eftimie Murgu Sq., 300041 Timisoara, Romania; 4 Public Health and Management Department, “George Emil Palade” University of Medicine, Pharmacy, Science, and Technology of Targu Mures, 540139 Targu Mures, Romania; 5 Faculty of Medicine, “George Emil Palade” University of Medicine, Pharmacy, Sciences and Technology, 540142 Targu Mures, Romania; 6 Department of Anatomy, “George Emil Palade” University of Medicine, Pharmacy, Sciences and Technology, 540142 Targu Mures, Romania; 7 Department of Pharmaceutical Technology, “George Emil Palade” University of Medicine, Pharmacy, Sciences and Technology, 540142 Targu Mures, Romania; 8 Close Circuit Pharmacy of County Clinical Hospital of Targu Mures, 540072 Targu Mures, Romania; 9 Department of Pathophysiology, “George Emil Palade” University of Medicine, Pharmacy, Science, and Technology of Targu Mures, 38 Gheorghe Marinescu Street, 540142 Targu Mures, Romania; 10 Department of Pathology, Akdeniz University School of Medicine, Antalya Pınarbaşı, Konyaaltı, 07070 Antalya, Turkey; 11 Department of Cardiology, “Victor Babes” University of Medicine and Pharmacy, 2 Eftimie Murgu Sq., 300041 Timisoara, Romania; 12 Department of Obstetrics and Gynecology, “George Emil Palade” University of Medicine, Pharmacy, Sciences and Technology, 540142 Targu Mures, Romania; 13 Department of Medical Informatics and Biostatistics, “Victor Babes” University of Medicine and Pharmacy, 2 Eftimie Murgu Sq., 300041 Timisoara, Romania

**Keywords:** dysgerminoma, OMGCTs, platinum-based therapy, heterogenous tumors, epigenetics, primitive germ cell tumors

## Abstract

Dysgerminoma represents a rare malignant tumor composed of germ cells, originally from the embryonic gonads. Regarding its incidence, we do not have precise data due to its rarity. Dysgerminoma occurs at a fertile age. The preferred treatment is the surgical removal of the tumor succeeded by the preservation of fertility. Even if a multidisciplinary team, founded in 2009 by a gynecologist, an oncologist, a pediatric oncologist and a pediatric surgeon, under the guidance of the Malignant Germ Cell International Consortium (MaGIC), studies this type of tumor, issues still remain related to the lack of a randomized study and to both the management and understanding of the concept of OMGCTs (ovarian malignant germ cell tumors). The aim of this review is to present from the literature the various approaches for this type of tumor, and, regarding innovative therapies or possible prevention, which can be applied in clinical practice. Multidisciplinarity and treatment in reference centers have proven their usefulness as well.

## 1. Introduction

Dysgerminoma is a malignant tumor composed of germ cells histogenetically derived from the embryonic gonads, known as the equivalent of testicular seminoma, according to the World Health Organization (WHO) [1,2]. The most common type of nondysgerminomatous tumors are immature teratomas, endodermal sinus tumors (yolk sac), embryonal carcinomas, polyembryomas, choriocarcinomas and mixed germ cell tumors [2]. Tumors with primitive gonadal cells are histologically characterized by their development from primitive germ cells, which does not have a specific pattern of differentiation [3].

It is a rare tumor, most often originating from a dysgenetic gonad, with the presence of a Y chromosome. Among gonadoblastomas, dysgerminoma is the most common [4], first described by Scully et al. as a rare tumor with unknown prevalence [5]. It can be frequently associated with hermaphroditism, being first described by Swyer et al. in 1955 in a hermaphrodite with a 46XY karyotype and a female phenotype [6]. It occurs more frequently in adolescence/young adulthood [1,2], representing 1–2% of all ovarian neoplasia [2,7]. The etiopathogenic mechanism is not yet completely known [3]. It is one of the malignant germ cell tumors (OMGCT), which are heterogeneous tumors derived from the primitive germ cells of the embryonic gonads, and is rare, representing 2.6% of malignant ovarian tumors [8]. Dysgerminoma is the most frequent [8]. Regarding its incidence and epidemiology, we do not have precise data due to the rare condition of the disease [9]. It is discovered more frequently in stage I, according to FIGO (International Federation of Obstetrics and Gynecology) staging, in a proportion of 75% [2,7]. These tumors are usually unilateral, but can also develop bilaterally, usually not accompanied by ascites [7].

Dysgerminoma occurs at fertile age, and we can find it in association with 2.8-11/100,000 of pregnant women [10,11,12]. In the literature, there are case reports presented with misdiagnosed dysgerminomas and confused with fibromas, including by ultrasound and MRI examinations. It is usually discovered during the caesarean section performed due to a dysfunctional labor caused by this previa tumor [13]. The follow-up also includes a PET (positron emission tomography) scan [10,13]. A multidisciplinary team formed by a gynecologic oncologist, a pediatric oncologist and a pediatric surgeon, under the guidance of the Malignant Germ Cell International Consortium (MaGIC) and founded in 2009, studies this type of tumor while important organizations such as the European Society of Gynecology Oncology (ESGO) and European Society for Pediatric Oncology (SIOPE) define the standards for diagnostic, treatment and follow-up [9].

The favorite treatment is the surgical removal of the tumor and the preservation of fertility [2,7,10,13,14], but, in the case of hermaphroditism, mixed germ cells tumor can develop, which leads to a more aggressive evolution, with a risk of malignancy in the bilateral of the gonads, which is why removal of both ovotestis is required [15]. Recurrence reaches 20% in 2 years, after being successfully treated by adjuvant methods, chemotherapy with platinum-based therapy with good efficiency and tolerability and, rarely, radiotherapy [7].

The prognosis is excellent after surgical treatment, often with the preservation of fertility [7]. However, issues still remain related to both management and understanding, regarding the concept of OMGCTs (ovarian malignant germ cells tumors).

## 2. Materials and Methods

We systematically searched PubMed and Medline databases for the literature published from January 2012 to July 2022. The inclusion criteria were all English-written literature related to dysgerminomas and ovarian germ cell tumors, as the main subject, including seminomas and extragonadal germ cell tumors. Most of the manuscripts were case reports, due to the rarity of the disease.

## 3. Diagnosis

Symptoms of this disease are pelvic pain, tumoral mass with abdominal distention, amenorrhea, sometimes bleeding and compression of the neighboring organs, but it can also be asymptomatic, as in our case [2,7,9,13]. However, the main symptom is represented by pelvic and abdominal pain, which can be acute, in the case of rupture, torsion or hemorrhage [7,11,16,17,18,19]. Family history is usually insignificant for this pathology [20]; moreover, the family history of cancer inversely correlates with the development of germ cell tumors [9]. Personal history is irrelevant as well [20]. The clinical diagnosis, along with the described symptoms, would be the general appearance of hermaphroditism in some specific cases and sexual ambiguity [21], palpable abdominal mass tumor, abdominal distension [6,9,11] or tumors in the inguinal region [21].

**Figure 1:**
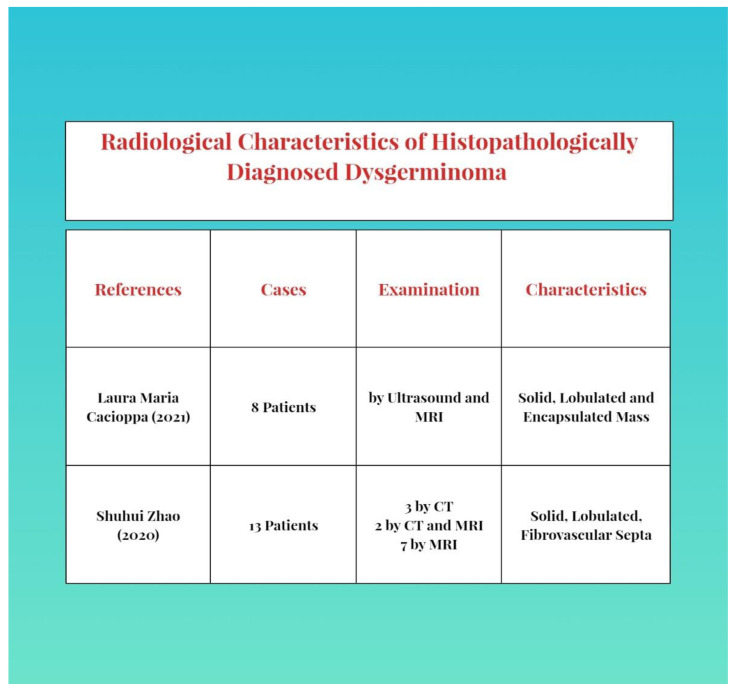
Characteristics and common features in examination. Refs. [23], [18] in order with the context

As imaging, we can use abdominal and more frequently transvaginal ultrasounds and the associated Doppler velocimetry [2,9]. The characteristics are a solid, multilobulated, heterogeneous tumor mass, separated from the uterus, with fibrous septa and an irregular appearance. We can find anechoic areas of necrosis or intratumoral hemorrhage. During a Doppler examination, we can a find a low resistance flow [2,3]. A similar aspect can be found in an MRI [8,9,18,22]. A CT scan is used less often, as an ultrasound and MRI are more specific for diagnosis [23]. The description of the aspects and common features of examination are presented as follows in Figure 1.

For detection of metastases and pleurisy, a chest X-ray would be useful [9]. Follow-up includes a PET scan (positron emission tomography) [9,10,13].

**Figure 2:**
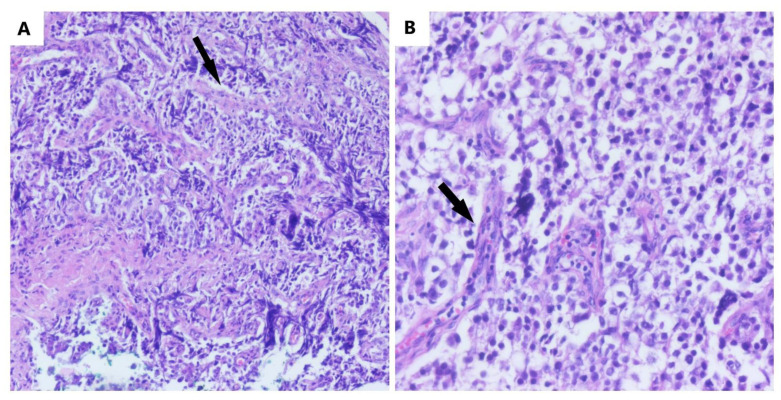
The microscopic aspect of dysgerminoma: (**A**) nests and nodules of uniform tumor cells, which are polygonal in shape, with clear-visible cell borders, an eosinophilic-to-clear cytoplasm and centrally located nucleus, separated by fine connective tissue containing inflammatory cells (black arrows) (HE, ob. 10×); (**B**) details of the described area (HE, ob. 20×).

A histopathological examination establishes the final diagnosis. The macroscopy reveals gray-whitish tumors, which are encapsulated and rarely bilateral and sometimes have areas of necrosis and hemorrhage [21]. The microscopic aspect is characterized by the nests and nodules of uniform tumor cells, which are separated by fine connective tissue, containing inflammatory cells. The tumor cells are polygonal in shape, with clearly visible cell borders, with an eosinophilic to clear cytoplasm that is centrally located, with a round nucleus and prominent nucleoli (Figure 2).

It can be admixed with cord derivatives, such as Sertoli or granulosa cells. Calcifications can also be present. Ovarian stromal cells only appear focally. It can be mapped to the Y chromosome, which could be detected, and to the 12 chromosome’s p arm [24,25]. In addition, for a complete and correct diagnosis, especially in the case of hermaphroditism, the determination of the karyotype is requested, possibly with a buccal swab for the SRY gene mutation as well [5,6,20,26]. In addition, in gonadoblastomas, it would be useful to search for DNA sequencing and for the status of the 12 chromosome’s p arm from the tumor cells, by cytophotometry and fluorescence in situ hybridization (FISH), especially since this is associated with reserved prognosis and additional therapy [24]. Valuable biomarkers for the diagnosis, differential diagnosis and follow-up of post-therapeutic evolution are BHCG (beta-human chorionic gonadotropin) [2,4,9], LDH (lactate dehydrogenase) [2,4,10,13] and AFP (alpha-fetoprotein) [13], which can be negative [3]. For a differential diagnosis with other ovarian tumors, cancer antigen 125 (CA-125) can be used [9]. The differential diagnosis is made with early or ectopic pregnancy, due to the presence of BHCG [4,27], uterine fibroids [10], other forms of acute abdomen [10], lymphoma or leukemia in case of atypical skin or breast metastases [28] and endometriosis [14].

Dysgerminomas can complicate by rupture, torsion, hemorrhage or incarceration [9,10,11]. Either they can give local metastases (neighboring tissue), via lymphatic, or distant hematogenous metastases in bones, lungs, the omentum, kidneys [3,8], the breast and skin (rarely but aggressively) [12,13] or the neck [14]. Confirmation diagnosis for germinoma metastasis is made by histopathological examination using immunohistochemistry [14,28].

## 4. Management

Management in the case of this diagnosis would be primarily surgical. Minimally invasive interventions are preferred. Sometimes laparoscopy does not detect pathological lesions [27]; in this case, biopsies are performed. If there are changes suggestive of hermaphroditism, we prefer the removal of the gonads in the first attempt or in two stages, due to the malignancy capacity of the ovotestis [3,6]. In these situations, most of the time, the gonads are found in the inguinal canal [6]. However, in the case of typical dysgerminoma, we prefer a unilateral oophorectomy with the preservation of fertility [2,9,10,13,14]. Tumor cytoreduction is followed by the best survival [8,17]. Multiple peritoneal and epiploic biopsies are associated only if we find abnormalities [8], with peritoneal washings preferred [9]. In some complicated situations, a hysterectomy was also required [29], though only in an advanced stage (II or III), sometimes with a lymphadenectomy [8]. In some cases, these extensive interventions are even abandoned when fertility preservation is desired [8]. In some studies, lymphadenectomy and omentectomy are even recommended as the first intention [19], but this is not supported by the majority of studies [7,8,17]. The postoperative outcome is adequate. Postoperatively, adjuvant chemotherapy may or may not be required [7]. For example, platinum-based therapy [8] can improve the prognosis [6] with a survival of 100% in early-stage tumors and 75% in advanced-stage tumors [8]. After gonadal removal, a hormone replacement therapy would be necessary [6,29]. Second-look surgery seems to have not demonstrated its usefulness, if there is no tumor recurrence or metastases or the biomarkers have not increased [8].

If diagnosed during pregnancy, it would be most appropriate to remove it surgically in the second trimester of pregnancy, at 16–18 weeks, followed by adjuvant chemotherapy, either during pregnancy or after birth, depending on the intraoperative findings [10,11]. The survival rate is between 97–100%, with the preservation of fertility [2,10,11]. Even if diagnosed in advanced stages, the survival rate is over 80% [11]. The solution, in case of gonadal removal, is represented by the assisted human reproduction technique, egg donation and embryo transfer [29].

## 5. Recurrence, Prognosis, Metastasis and Follow-Up

**Figure 3:**
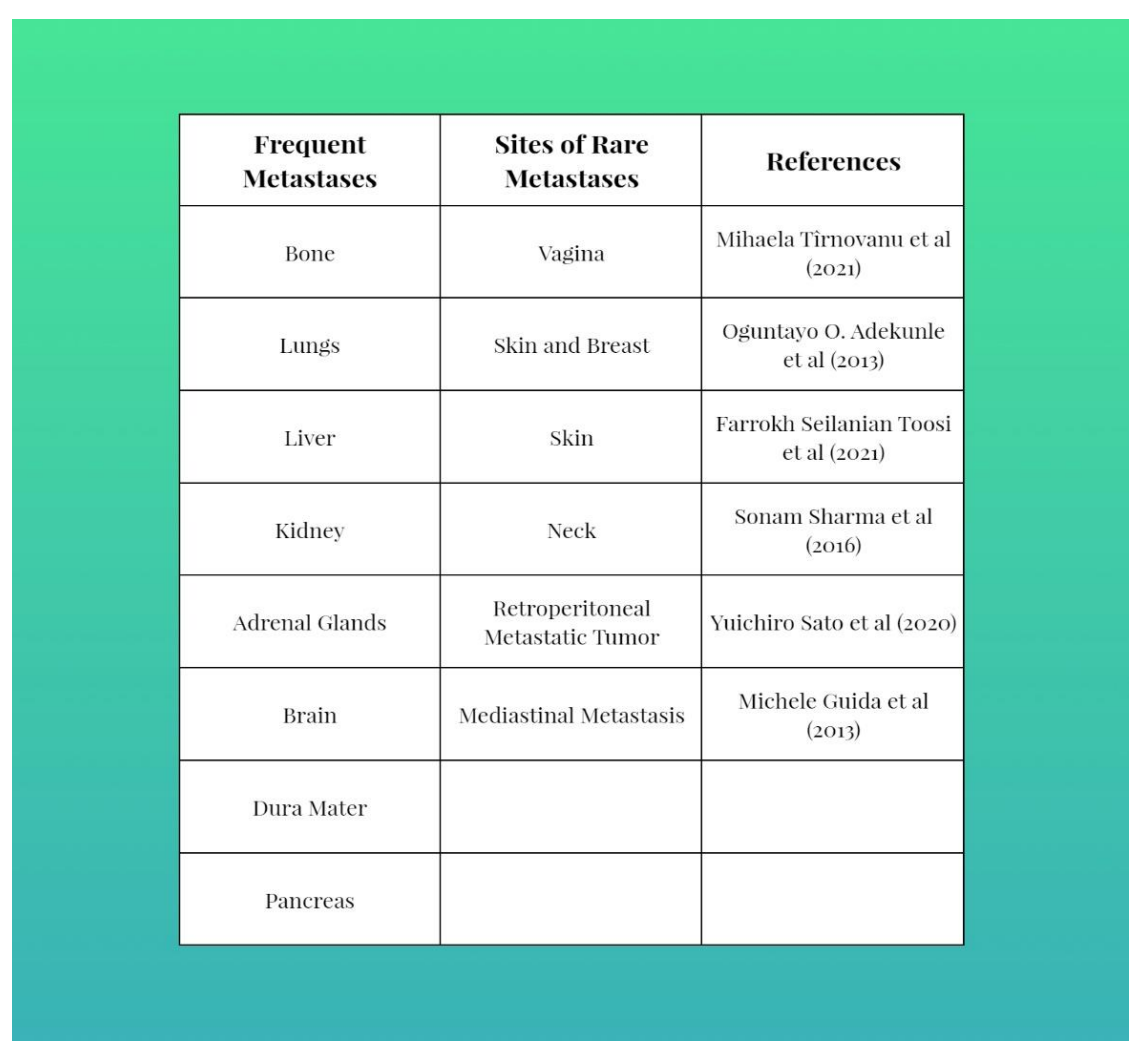
Frequent versus rare metastasis sites. Refs. [3], [28], [14], [31], [5], [30] in order to the context

Recurrence reaches 20% in 2 years and is successfully treated by adjunctive methods, chemotherapy with platinum-based therapy with good efficiency and tolerability and, rarely, radiotherapy [5,7,9,14,30]. This type of tumor can spread peritoneally, lymphatically and distantly, in bone, lungs, liver and brain (Figure 3). In case of metastasis, these tumors respond to chemotherapy with bleomycin, etoposide and cisplatin [31]. Tee notable side effects of chemotherapy are thrombocytopenia and fever [32]. In the case of tumors that do not respond to chemotherapy, in a proportion of 10–15%, immunotherapy is applied as additional treatment to inhibit the reaction between PD-1 and its ligand PDL-1 (programmed death receptor and its ligand) [33].

A poor prognosis is influenced by the stage, histology, grading, association with other germinal tumors such as the yolk sac, age over 45 years at the time of detection, association of some genetic anomalies, values of tumor biomarkers, residual tumors after cytoreduction and treatment administered outside the reference centers [8,9]. A comparison between various studies, regarding the diagnosis and therapeutic approaches, is presented in Table 1.

We chose to structure Table 1 in this way because it was the only able way to compare the studies, while considering the heterogeneity of the evaluated data by their authors and its variety in management. If the majority preferred a fertility-sparing surgery with USO (unilateral salpingo-oophorectomy), as less aggressive as possible, our colleagues from Turkey used a lymphadenectomy and an omentectomy in all cases, as can be seen from Table 1. So the varied approaches require the implementation of a coherent strategy. In addition, we showed there are differences between adult oncology and pediatric oncology, for which we have not yet found a solution.

A case with a severe evolution, which draws attention, is the aggressive systemic mastocytosis (ASM) complication of dysgerminomas, an extremely aggressive and rare form of cancer, with an accumulation of mast cells in various organs that can derive from dysgerminomas. The etiological link can be seen after cytogenetic molecular analysis, with the KITD8 16A variant, which is the same as the one discovered in dysgerminomas by histopathological examination [1]. This fact highlights the need for further study of genetic mutations, which can help to evaluate the prognosis and the management of the disease. Another disease with a fulminant evolution, resulting in the patient’s death, is HLH (hemophagocytosis lymphohistiocytosis), a rare form that evolves with pancytopenia and in association with dysgerminomas, becoming fatal [6].

A follow-up is necessary once at 3 months in the first 3 years and at 6 months in the next 2 years, followed by an annual evaluation up to 10 years [10]. However, relapses were mentioned in the literature at 39 years [2,5]. In addition, the usefulness of an annual evaluation is preserved even later, in the form of a long-term follow-up [5], which should include a clinical examination, an abdominal and pelvic ultrasound, an evaluation of biomarkers, in particular beta-HCG and LDH, AFP and CA-125, and a chest X-ray for pulmonary metastases. Moreover, pregnancy is encouraged as well.

The clinical approach for this pathology, therapeutic management and follow-up is exemplified in Table 2. Here, we present a synthesis based on other studies and what is known about dysgerminomas, all of the therapeutic elements and the management of diagnosis.

## 6. Other Ovarian Malignant Germ Cell Tumors, Differentiation and Management

Heterogenous tumors derived from germinal tumors of the embryonic gonads, which in order of frequency of occurrence are as follows:

▪ dysgerminomas;
▪ immature teratomas;
▪ yolk sac tumors;
▪ polyembryomas;
▪ mixed germ cell tumors;
▪ embryonic carcinomas;
▪ choriocarcinomas;
▪ struma ovaries.

The incidence is 0.34–0.41 per 100,000 women according to the WHO, the most common being dysgerminoma [8]. In diagnosis and during follow-up, the mentioned biomarkers help, but in dysgerminoma their presence may be missing. Management, even if it is not systematized, due to the heterogeneity of these types of tumors, is the most successful among ovarian neoplasms, ensuring a 5-year survival rate of 100% in the case of dysgerminomas and 85% in the case of other germinal tumors, if discovered in the early stages [8].

Malignant germline tumors represent a heterogeneous class that, in many cases, can contain several elements, which are able to modify the prognosis and prevent a coherent histological grading. Thus, the association of a yolk sac tumor with dysgerminoma worsens the prognosis. There are associations with seminomas, carcinoids and elements of mature or immature teratomas, which is why it is important to describe them well, in order to differentiate and identify their various histological components. This fact is useful not only for a correct and complete diagnosis but also, especially, for therapeutic management. This amalgam of associations further complicates a rare pathology that is difficult to frame anyway. We chose to review the possible associations [9,18,19,34,35,36].

### 6.1. Yolk Sac Tumors

A yolk sac tumor is an endodermal sinus tumor, the third in frequency among OMGCTs. If it is associated with ovarian epithelial carcinoma, the prognosis worsens significantly [34]. It occurs in young women and represents 14–20% of malignant OMGCTs [34]. It can also appear extragonadal [35]. The macroscopic appearance is that of a large, encapsulated tumor, reaching over 15 cm, with a smooth surface and cystic areas alternating with solid, gray-yellow areas. It can present capsule ruptures, hemorrhages and necrosis or a honeycomb appearance that is suggestive for neoplasia. Microscopically, it is composed of structures with yolk cell elements originating from the endoderm sinus and contains benign elements. Imaging is characterized by a heterogeneous appearance with solid areas, cysts, rich vascularity, necrosis and hemorrhage [8]. BHCG and alpha-fetoprotein are secreted by the tumor and are used as biomarkers. Surgery associated with adjuvant chemotherapy is chosen as the therapy. Omentectomy with a total hysterectomy with bilateral oophorectomy and lymph node dissection did not improve the prognosis [36]. The explanation of the pathogenesis of this tumor is unknown [34].

### 6.2. Immature Teratomas

Immature teratomas are third in frequency among tumors with germ cells, are formed by three germ cell layers, ectoderm, mesoderm and endoderm, with at least one showing elements of immaturity, and were first described by Norris et al. in 1976 [37]. According to the WHO classification, ovarian teratomas fall into three categories: immature, mature (cystic or solid) and monodermal highly specialized. The most common are mature cystic teratomas, under the name of dermoid cysts [38]. Immature teratomas resemble mature teratomas in appearance, though the prognosis is worse. Macroscopically, they reach large dimensions of over 25 cm and are encapsulated, predominantly solid and smooth, with cysts full of mucinous, fatty, sebaceous fluid. The surface is multinodular, brown, pink and gray. Immature teratomas present areas of necrosis and hemorrhage, containing hair, fat, teeth and sebaceous material. This aspect can also be found in the contralateral ovary. Microscopically, they consist of three germ layers and a mixture of mature and immature elements.

The grade of the tumor depends on the concentration of the immature neural tissue contained in the tumor and has prognostic value [39]. The imaging is non-specific, similarly for a heterogeneous ovarian tumor mass, which is predominantly solid with tooth-like calcifications and fatty, sebaceous cystic content. In certain situations, a PET scan is useful for the preoperative detection of lymph nodes, in the case of metastases at their level, but the diagnosis is made by the biopsy [40]. BHCG and alpha-fetoprotein are secreted as biomarkers. Due to its worse prognosis, conversion to a mature teratoma through chemotherapy, followed by surgery, can be chosen as the therapeutic management [8]. Although the proposed management is surgical with the preservation of reproductive capacity, 80% of patients attempting to conceive become pregnant [41]. Even if the tumor is discovered in the advanced stages, fertility can be preserved if the tumor cytoreduction is performed correctly at the first surgical intervention [42]. In pediatric oncology, only this method is used, without the combination of adjuvant chemotherapy as tumor management [41]. In the case of recurrence, the management is still not established due to the lack of data from the literature [41]. Thus, the management in pediatric oncology is different from that in adult oncology, where we can encounter late recurrence. The follow-up period is not standardized either [41,43].

#### 6.2.1. Malignant Struma Ovarii

A malignant struma ovarii is a rare, less-known tumor, which is part of a mature cystic teratoma, with malignant areas formed by thyroid tissue. Many are asymptomatic, with the only manifestation being hyperthyroidism. If it appears as a pelvic tumor mass, it is macroscopically unilateral, with amber-colored colloid compartments. Microscopically, it shows mature thyroid tissue with acini filled with colloid, and the malignant component contains follicular carcinoma and is anaplastic, with Hurthle cells.

Imaging is not useful in diagnosis, as it rarely appears as a solid mass with calcifications, ascites or pseudoMeigs syndrome [8].

#### 6.2.2. Malignant Carcinoid Tumors

Malignant carcinoid tumors are monodermal teratomas differentiated toward argentaffin cells, usually with a good prognosis. Macroscopically they have a solid aspect, are yellow colored and are unilaterally developed. Histologically, they show small acini, solid nests and uniform polygonal cells with abundant cytoplasm with argentaffin silvery granules. Imaging shows multilocular cysts with solid areas of sponge-like appearance [8].

### 6.3. Nongestational Choriocarcinoma

A nongestational choriocarcinoma is the rarest of the GMOCTs. The initial diagnosis is made by the increased BHCG biomarker, which is often confused with an ectopic pregnancy because of its occurrence at reproductive age. In the case of tumors, it has a significantly higher serum level than a pregnancy [44]. It occurs either from gonadal tissue or from the pure germ cells of the ovary with the development of nongestational choriocarcinoma. It represents only 0.6% of ovarian tumors with germ cells, and the incidence in the population is 1/369,000,000 [45]. The differential diagnosis is, of course, made with ectopic pregnancy or abortion [46]. As symptoms, patients complain of nausea, pelvic pain or, sometimes, vaginal bleeding [45]. Sometimes, it presents anemia, through multiple episodes of massive bleeding, with the tumor erodating the vascular walls [47]. Sometimes, it is associated with an ovarian hyperstimulation syndrome, due to the huge values of BHCG [47]. It is often detected when it metastasizes in the lungs or brain [45], which occurs in about one-third of cases [47]. Macroscopically, nothing specific is observed at the level of the ovary, and also nothing is observed by imaging, which rarely shows highly vascularized solid tumors. Sometimes, a pseudoMeigs syndrome with pleurisy is detected on a chest X-ray [45]. The diagnosis is microscopic, through a giant syncytial cell resembling the gestational one, only it develops primarily in the ovary [8]. Paternal DNA it is necessary to be determined from the biopsied tissue for differential diagnosis, as it is exclusively present in gestational tumors [46,48]. It responds less to chemotherapy compared to the gestational one. However, fertility-saving surgery with a combination of adjuvant chemotherapy is taken into account. There is no consensus regarding lymphadenectomy [49]. We could say that there is no consensus regarding the entire management of the disease, as it is extremely rare. It was demonstrated that fertility could be preserved. The pregnancy and birth rates were satisfying in the case of unilateral oophorectomy, but, on the other hand, in those with advanced disease, the prognosis is poor. Cytoreduction improves the prognosis, if the diagnosis is made in the presence of metastases. The response is significantly better if we administer adjuvant chemotherapy to reduce the size of tumors with high doses of platinum-based chemotherapy before cytoreduction [50]. Beta-HCG is also used in the evaluation of the therapeutic response [46].

### 6.4. Embryonal Carcinoma, Polyembrioma and Mixed Germ Cell Tumors

Embryonal carcinoma, polyembrioma and mixed germ cell tumors are very rare tumors, which occur in adolescence or young adulthood. Macroscopically, they present a smooth surface, which is gray-yellow in color, with areas of hemorrhage and necrosis, and are solid with cysts containing mucinous material. The values of BHCG, AFP and LDH biomarkers are increased. Microscopically, nests of large primitive cells in a pseudoglandular pattern, possibly with a mixed texture, can be seen. Polyembryoma, a very rare tumor in teenagers, is a mixed germ cell tumor, which secretes beta-HCG and AFP. Microscopically it consists of small embryo-like bodies with central “germ disks”. Frequently, it contains components from dysgerminoma, a yolk sac tumor with aggressive expression. Due to its rarity, we do not have an imaging description, and it is a histological surprise; for this reason, a quick and correct diagnosis is essential [8,51].

### 6.5. Ovotestis with Seminoma

An ovotestis with seminoma is the homologous variant of dysgerminoma, and two-thirds of people with ovotestis and seminoma are men, so it is extremely rare in gynecology. The location of the ovotestis can be lateral when a testicle is on one side and an ovary on the other (30%); bilateral with a testicle and ovary on both sides (50%); or unilateral ovotestis on one side and a testicle or ovary on the other (20%). The diagnosis is by laparoscopy with a biopsy and histopathological examination, consisting of a scattered tumoral pattern, which is interrupted by fibrous septa, with uniform tumoral cells represented by a clear-to-eosinophilic cytoplasm and a round-to-oval nuclei, with a finely granular chromatin and flattened edges. Chromosomal mapping of the histological piece would be important [34,52,53]. The seminoma can overlap a gonadoblastoma, which is developed from dysgenetic ovaries and includes a mixture of germ cells with immature Sertoli cells and granulosa cells and was first described by Scully et al. in 1970 [53].

As in dysgerminoma, biomarkers such as BHCG, AFP, LDH and alkaline phosphatase are important. Imaging can reveal ovotestis with seminoma inside the abdomen or groin region, with similar characteristics to a dysgerminoma [52,53,54,55,56]. It responds well to radiotherapy and chemotherapy with platinum-based products, but the first line of treatment is still surgical removal, due to malignancy.

The evolution and the prognosis are as good as in dysgerminoma, though psychological counseling has a big importance, and we refer to DSD disorders of sex development. There is no coherent management in this sense, as an extremely rare pathology, with the incidence being 1/100,000 newborns. Multidisciplinarity in treating the disease is extremely important, involving a urologist, an oncologist, a gynecologist, an endocrinologist, a radiologist, a pathologist, a geneticist, a laboratory doctor, a psychologist, a pediatrician and a psychiatrist [52,53,56].

Regarding the preservation of fertile function, it depends on the presence of a sufficiently developed uterus to allow for the development of a pregnancy; in this case, after the removal of the gonads, assisted reproductive techniques with egg donation, embryo transfer and hormone replacement therapy are used [54,57]. Moreover, the tumor can be detected during a caesarean section, not interfering with fertility itself as much as with the normal progression of labor [56].

### 6.6. Extragonadal Germ Cell Tumor

An extragonadal germ cell tumor is an uncommon neoplasm, with another anatomical location than the gonads. The pathogenesis is unknown, as we do not have enough data regarding if it is a primary tumor or an occult metastasis of a gonadal tumor. It represents a unique entity that behaves differently from the gonadal one, even if both have the same histology. That is why the correct diagnosis is essential for tumor management. These tumors can be anything: dysgerminomas, seminomas, yok sac tumors, etc. They appear more frequently in men, usually on the midline of the body or in the brain, mediastinum, retroperitoneum or coccyx. The most well-known is the sacrococcygeal teratoma, which is congenital. Etiopathogenesis is related to its development from precursor germ cells, erroneously arrested in median migration during embryogenesis [58].

## 7. Epigenetics, a Scientific Reality?

As a study idea, applied in the case of germ tumors in men, the role of genetics and environmental factors can be studied because the human model of germ cell tumors is not initiated by somatic mutations but by an epigenetic status. Germ cell tumors are not a simple class of neoplasms, they are a heterogeneous group, which can develop both in the gonads and extragonadally. They, thus, reflect a complex model of tumors in development, with each subclass showing peculiarities and a specific epidemiology. This aspect has been studied in men [59,60]. The genetic risk factors that can be involved are family history, monozygotic twins and polygenic risk. In addition, those from the environment have perinatal factors involved, and, in testicular tumors, a link was also found with birth weight, mother’s parity and bleeding during pregnancy. We would consider exposure to hormones, both exogenous and endogenous, more important during pregnancy. It remains to be seen to what extent diets and physical exercise could influence the evolution of germinal tumors; the same is true for exposure to environmental factors, for example, agricultural workers with pesticides. All these factors can interact with genes. The primordial germ cells follow a process of maturation, proliferation and migration along the midline of the body, up to the gonad area, which explains the location of extragonadal tumors. Once they reach the genital area, we call them gonocytes, which will then differentiate into preoocytes, constitutionally dependent on the sex chromosome. The SRY gene plays a major role as does the gonadal microenvironment. DNA methylation also plays an important role here. Parental-specific DNA methylation has a genetic role in the differentiation of gonadal cells. A full understanding of all the stages of embryonic development helps us to conceive a unifying model for the pathogenesis of OMGCTs. Studies with epigenetic biomarkers are needed to be used in clinical practice [59]. It would also be useful to study the use of a microRNA oncogene as a biomarker [9].

## 8. Coherent Management Guidelines—A Necessity

Multidisciplinarity is important, and the management recommendations are few. A trial based on data from the literature cannot be conducted, as no model is reproducible. FIGO and NCCN guidelines recommend a complete staging procedure for OMGCTs that includes omentectomy, lymphadenectomy, peritoneal biopsies and washing for the management of epithelial tumors. However, this is questionable. Especially, the importance of lymphadenectomy in the management of OMGCTs is controversial. This is because it failed to demonstrate a survival benefit for women. The majority of women with this kind of tumor have not completed their fertility potential. Reproductive-sparing surgery should be used, even in the case of disseminated disease. The reported pregnancy and birth rates after treatment are encouraging. Platinum-based multi-agent chemotherapy is recommended by both the National Cancer Network and European Society of Medical Oncology (ESMO) and has become a standard for all OMGCTs treated in referral centers [36].

In their guidelines, MaGIC and ESGO recommend, as the standard therapy in stage IA, surgery alone, while in IB and IC chemotherapy is recommended. After surgery and in all other stages of tumors, surgery followed by prompt chemotherapy is the proper therapy. However, there is an inconsistency between ESGO and SIOPE, which consider that chemotherapy in Stage IB and IC is not recommended in pediatric oncology, only active surveillance, because of its toxicity. So an important issue is BEP long-term toxicity and how to reduce it. Another problem is that, even if ESGO does not recommend lymph node dissection in every stage of disease, Cigen Kilic et al. performed this procedure in every surgery they made, according to their study from 2021 [19]. Thus, correct management is still an issue.

The treatment should be standardized, and primary surgery with unilateral salpingo-oophorectomy with fertility preservation is the gold standard for this type of tumor in stage I, but, in case of hermaphroditism, it is necessary to remove both gonads due to the risk of malignancy [9,17]. The need for therapy and follow-up in reference centers where patients benefit from multidisciplinarity, thus, becomes the target pursued in the management of these tumors.

Even in the case of advanced tumors, in which surgical intervention with a loss of fertility followed by chemotherapy is initially indicated, chemotherapy followed by fertility-sparing surgery is proposed with the same results and a 5-year survival prognosis of 60–80% [32,60]. The evolution of dysgerminoma’s treatment is one of the most successful in the history of gynecological oncology [9]. This is also the recommendation of the ESME—European Society of Medical Oncology [9]. Since the introduction of platinum-based therapy in the 1980s, the 5-year survival rate in the cases in stage I has reached 100%, and the 5-year survival rate of advanced cases has reached 98% [9]. Adjuvant therapy focuses on reducing toxicity and long-term adverse reactions with the involvement of multidisciplinarity: pediatric surgery, pediatric oncology, gynecology, gynecological oncology and, of course, pathological anatomy [9]. The optimal cycles of chemotherapy are not yet fully known [32]. The ESGO recommendation has been related to five cycles of BEP [9] until now, but there are still controverses.

Regarding the staging, it is also questionable, due to the mixed cellularity encountered [23]. In the literature, difficulties are faced as well [19].

Regarding future concerns, we can mention immunotherapy for those who develop a resistance to chemotherapy, targeted against PD-1 and PDL-1 [33], or chemotherapy, followed by stem cell transplantation, in the case of a relapse [9].

## 9. Conclusions

Dysgerminoma is a malignant tumor with an excellent prognosis. The evolution of dysgerminoma’s treatment is one of the most successful in the history of oncological gynecology, and the preservation of fertility should be considered the gold standard. Lymphadenectomy and omentectomy should not be part from the first-line therapy. Management needs improvements as well, and a consensus between pediatric oncology and gynecological oncology currently represents an unreached goal.

Regarding innovative therapies, immunotherapy is already used, while targeted therapy has not yet brought any benefit. Prevention should be given more importance, and, in this light, studying epigenetics would be the next future step. Moreover, multidisciplinarity and treatment in reference centers have proven their usefulness.

## Figures and Tables

**Table 1:** Comparison between various studies, regarding the diagnosis and therapeutic approaches.

All Tumors Diagnosed by Histology							
References	Stage	Main Symptom	Therapeutic Management	Relapse	Five Year DFS (Disease Free Survival)	Five Year OS (Overall Survival)	Fertility Preservation
Hamid Al-Huseini et al., 2012 [7]–65 patients	▪ 21 pt. I A (32.3%); ▪ 12 pt. I C (18.5%); ▪ 2 pt. II A/IIB (3%); ▪ 4 pt. III A/IIIB (6.2%); ▪ 18 pt. III C (27.6%); ▪ 4 pt. IV (6.2%)	Pelvic pain (most frequent) 44 pt. (67.7%); Asymptomatic 4 pt. (6.2%)	Surgery BO 4 pt. (6.2%); UO 50 pt. (76.9%) Post-Surgery Adjuvant Therapy ▪ CHT 40 cases (61.5%); ▪ RT 4 cases (6.2%); ▪ NT 21 cases (32.3%)	6 pt. (9.2%)	88%	100%	Out of 50 patients treated with fertility-sparing surgery, 16 patients (32%) became pregnant
Antonio Bandala-Jacquer et al., 2019 [17]–180 patients ▪ 166 pure dysgerminoma (92.1%); ▪ rest presented mixed histology	▪ 80 pt. (44.4%) unstaged; ▪ 54 pt. (30%)–I; ▪ 9 pt. (5%)–II; ▪ 29 pt. (16%)–III; ▪ 8 pt. (4.4%)–IV;	Pelvic pain *112 pt. (62.2%)*	Surgery 71 pt. (39.4%) from which 51 (28.3%) TAH.; 14 pt. (7.8%) BO; 32 pt. (17.77%) LnD; Adjuvant therapy 135 pt. (75%) ▪ CHT 125 cases (69.3%); ▪ RT 13 cases (7.2%)	4 cases (2.2%) pelvic; 6 cases (3.3%) nodal; 3 cases (1.7%) distant	92.8%	97.9%	37 pt. (20.6%) became pregnant
Cigen Killie et al., 2021 [19]–18 patients	▪ 6 pt.-I A; (33%) ▪ 4 pt.-I C; (22%) ▪ 1 pt.-II A; (5%) ▪ 1 pt.-II B; (5%) ▪ 1 pt.-III A; (5%) ▪ 4 pt.-IIIC; (22%)	Pelvic pain 7 pt. (38%)	Surgery UO 13 pt. (72%); BO 1 pt. (5%) THBSO 4 pt. (22%) LnD 18 pt. (100%) Adjuvant Therapy ▪ CHT 8 pt. (44%); ▪ RT 1 pt. (5%) ▪ CHT and RT 2 pt. (11%)	3 pt. (16%)	Not Applicable (Follow-up in progress)	Not Applicable (Follow-up in progress)	2 cases (11%)

pt.—patients; BO—bilateral oophorectomy; UO—unilateral oophorectomy; CHT—chemotherapy; RT—radiotherapy; NT—neoadjuvant therapy; TAH—total abdominal hysterectomy; LnD—lymph node dissection; THBSO—total hysterectomy and bilateral salpingo-oophorectomy.

**Table 2:** Diagnosis and therapeutic approach based on various studies.

Family History	Personal Medical History	Symptoms	Clinical Diagnosis	Imaging US, MRI, CT, PET Chest X-Ray	Biomarkers	Histopathology	Management	Prognosis	Follow-Up
Family cancers are inversely linked with this type of tumor	NAD	NAD	NAD	NAD	β-HCG	Microscopic changes	Fertility-sparing surgery	Histopathology	Every 3 months/ 3 years
		Pelvic pain	Tumoral mass	Tumoral mass	AFP	Chromosome mapping	Surgery (unilateral oophorectomy)	Age	Every 6 months/ 2 years
		Nausea	Bleeding	Calcifications	CEA	Buccal swab	BEP (chemotherapy)	Staging	Annually/ 10 years
		Bleeding	Casexia	Vascular changes	LDH	Peritoneal washing cytology and Biopsy	Bilateral oophorectomy and hormone replacement therapy	Grading	Duration of long-term follow-up is not established
	Amenorrhea	Metastases	Low-resistance flow (Doppler)			Total hysterectomy	Assoc. with other germinal tumors		
		Hermaphroditism	Hemorrhage			Omentectomy	Genetic anomalies		
			Necrosis	CA-125		Lymphadenectomy	Biomarker levels		
			Ascitis (rare)			Radiotherapy	Residual tumors		
			Pleuresia (rare)				Treatment outside reference centers		

NAD—nothing abnormal discovered; β-HCG—beta-human chorionic gonadotropin; AFP—alpha-fetoprotein; CEA—carcinoembryonic antigen; LDH—lactate dehydrogenase; CA-125—cancer antigen 125; BEP—bleomycin sulfate, etoposide phosphate and cisplatin.

## Data Availability

Not applicable.
